# Knowledge Extraction and Semantic Annotation of Text from the Encyclopedia of Life

**DOI:** 10.1371/journal.pone.0089550

**Published:** 2014-03-03

**Authors:** Anne E. Thessen, Cynthia Sims Parr

**Affiliations:** 1 Arizona State University, School of Life Sciences, Tempe, Arizona, United States of America; 2 National Museum of Natural History, Smithsonian Institution, Washington, District of Columbia, United States of America; Indiana University, United States of America

## Abstract

Numerous digitization and ontological initiatives have focused on translating biological knowledge from narrative text to machine-readable formats. In this paper, we describe two workflows for knowledge extraction and semantic annotation of text data objects featured in an online biodiversity aggregator, the Encyclopedia of Life. One workflow tags text with DBpedia URIs based on keywords. Another workflow finds taxon names in text using GNRD for the purpose of building a species association network. Both workflows work well: the annotation workflow has an F1 Score of 0.941 and the association algorithm has an F1 Score of 0.885. Existing text annotators such as Terminizer and DBpedia Spotlight performed well, but require some optimization to be useful in the ecology and evolution domain. Important future work includes scaling up and improving accuracy through the use of distributional semantics.

## Introduction

Biological knowledge, accumulated over centuries of observation and experimentation, is contained within the legacy format of printed text, which may or may not be digitized [1]. Because many observations are expensive or impossible to duplicate, Biology as a discipline, needs to leverage the scalability of computing to optimize use of all existing data. Because the volume of information is far more than any human can read in a lifetime, a key challenge for biology is migrating this vast amount of knowledge into modern, machine-readable formats so a computer can do the work of data discovery. Tools designed to aid in the manual annotation of text have improved the rate of digitization, but this process needs to be streamlined further if it is to catch up to the current rates of species discovery (approximately 19,000 new species every year, [2]). Approaches that involve generating marked-up manuscripts from databases are emerging [3] but so far only in taxonomy and not in ecology, and mark-up is not yet as detailed as needed.

Decades of research into Natural Language Processing and Machine Learning have resulted in a suite of freely available algorithms capable of extracting information from general text, such as newspaper articles [4]. The specialized nature of biological text requires the development of new algorithms or modification of existing algorithms [5]. Many specialized algorithms exist and continue to be refined for the purpose of identifying species names in text and extracting morphological information and molecule interaction information (See review of biodiversity related NLP tools in [5]). Machine learning is not yet advanced enough to allow large-scale extraction of knowledge from biological text without human input (at least not life-wide, see [6] for a tool that works well for plant descriptions). Numerous annotator tools have been developed to aid the process of human text markup to guide machines (see [7] for a biodiversity example).

In addition to using algorithms to extract information from text, placing data in machine readable formats and making it more interconnected increases the usefulness of data by making it easier to find and combine in novel ways by people other than the original collector [8]. The linked open data cloud (LOD) is one manifestation of this type of data mobilization [9]. There are over 31 billion triples in LOD and 9.6% of them are from life sciences data sets [9]. Many of these life sciences data sets have a molecular focus, but some, like TaxonConcept (http://datahub.io/dataset/taxonconcept) and GeoSpecies (http://datahub.io/dataset/geospecies) are biodiversity-related. Some members of the taxonomy community have embraced Semantic Web technology as a tool that can streamline the process of describing species, make optimal use of the data collected by taxonomists and efficiently manage species information [10]. RDF (Resource Description Framework), one of the languages of the Semantic Web, has been identified as a machine-readable way to express taxonomic information [11]. Organizations such as TDWG (Taxonomic Databases Working Group) and others have been working to develop a semantic framework for describing biodiversity information [12], [11], [13]. A major roadblock to developing large quantities of machine-readable data is widespread adoption of community standards, such as unique identifiers for species, specimens and observations and how to model such data. Proposed biodiversity semantic structures focus on describing nomenclature (TDWG), phenotype (PATO) and observations (TDWG and OBOE; [14]). The field of evolutionary informatics has recently been defined and strives to organize information about species around the structure of a tree of life [15]. The related field of ecoinformatics has developed a strong network of tools and support for ecological data throughout the data lifecycle [16]. Standard vocabularies for habitats exist and can be used to relate species to their habitats (for example, EnvO (http://environmentontology.org). Ontologies and schema treating ecological species associations have been attempted several times ([17]; (http://wiki.tdwg.org/twiki/bin/view/DarwinCore/InteractionExtension; SWEET (sweet.jpl.nasa.gov), SEEK (seek.ecoinformatics.org), ETHAN ([18]; bioportal.bioontology.org/ontologies/1530), SWISST (foodwebs.org), and TRIN (http://bit.ly/1cXHoHs), but a leading standard has not yet emerged. A complete semantic representation of biodiversity data will likely use components from all of these ontologies.

The biomedical community has fully embraced machine learning, informatics and semantics as a way to improve information sharing and discovery. Numerous tools exist for extracting molecular entities, diseases and interactions from text [19], [20]. Large standardized repositories exist for storing biomedical and molecular data (GenBank, uniprot, KEGG, ArrayExpress, OMIM, PubMed and BTRIS) that offer archiving, citation and visualization tools. The U.S. National Library of Medicine has developed the Unified Medical Language System, which functions as a Biomedical WordNet, providing standardized definitions for biomedical terms and linking related terms in a Semantic Network [21]. Ecoinformatics has not received the same level of investment as biomedical informatics, but both disciplines suffer from ambiguous language, use of abbreviations and the constant creation of new terms [22]–[24].

Biodiversity sciences are particularly crippled by data heterogeneity because of the distributed nature of data collection and the need to consider the entire body of knowledge [13]. Thus, a semantic infrastructure that improves data discovery and integration, has the potential to make a disproportionate, positive effect on advances in the discipline. There are thousands of databases and journals holding biodiversity information (too many to name all here) all with a different taxonomic and geographic scope. The advantages of using RDF, or some other semantic markup, to manage biodiversity data stem from the ability to assign unique identifiers (URIs) to names, taxa, concepts, etc. and then link information using those identifiers [11]. Much of the conversation has been dominated by linking data phylogenetically and taxonomically, but semantic structures also have the potential to link information based on interactions between taxa, between a taxon and its environment, and ultimately to look at ecological or environmental interactions in another context (phylogeny, for example). Building this semantic structure is non-trivial because many data sources use their own terms and identifiers; however, within biodiversity science, standards are being developed and followed by some major biodiversity data sources [25], [26].

The Encyclopedia of Life (www.eol.org) is a web site with the ambitious goal of creating a web page for every species and enabling global access to knowledge about life on earth. They are accomplishing this goal by being a content aggregator rather than authoring species pages [27]–[29]. EOL develops relationships with existing data sources to have their content featured on an EOL species page with full attribution and creative commons licenses (content partners include more than 250 museums, government agencies and research consortia). EOL has also developed a network of credentialed curators who label aggregated content as trustworthy or untrustworthy. All content featured in EOL can be accessed through an API that gives data in XML or JSON formats. Anyone with internet access can gain EOL content easily through this API.

The goals of this project were to develop and evaluate workflows to mine information held in EOL text objects in order to 1) annotate them with DBpedia URIs for biological entities relevant to EOL user interest, and 2) generate a global species association network. These two tasks, even at a very basic level of success, provide the foundational structure needed to explore EOL information from an ecological perspective rather than its current taxonomic perspective. Outside of EOL, these goals are a step toward large-scale analyses of biodiversity data based on ecological relationships. Since 1) there are approximately 2 million described species, 2) each species can have up to 100 text objects or more, and 3) EOL content is updated weekly from hundreds of content partners, any tool for interacting with EOL text must be dynamic, scalable and capable of handling heterogeneous structure. To demonstrate proof-of-concept, we have chosen 21 test species. In this paper we will describe and evaluate our workflows and the structure of the extracted data. We also provide pointers to data files (Appendix S1) and recommendations for further analysis.

## Materials and Methods

### Selection of test species

To explore approaches to achieving our two goals we chose 21 test species across the tree of life (Table 1). To limit overfitting, we chose species that are diverse in their taxonomy, life history, habitat, ecological niche and EOL content. Any process that operates over the entire tree of life will require attention to scalability. We have taken some basic measures to improve scalability within this project and discuss strategies to further improve scalability in future work (see discussion).

**Table 1:** Test Species and URI Annotation Results.

Common Name	Scientific Name	EOL Taxon ID	Number of Text Objects in English	Number of Correct URIs	Number of Correct and Unique URIs	Precision	Recall	F1 Score
Great White Shark	*Carcharodon carcharias*	213726	56	169	45	0.918	1	0.956
Lion	*Panthera leo*	328672	52	173	45	0.961	1	0.980
Moss	*Hypnum fauriei*	861365	2	0	0	0	0	0
Diatom	*Pseudo-nitzschia australis*	904440	19	23	14	0.852	1	0.92
Mosquito	*Culex pipiens*	740671	8	8	6	1	1	1
Bacteria	*Escherichia coli*	972688	7	36	17	0.783	1	0.878
Cactus	*Harrisia simpsonii*	594934	20	13	9	1	1	1
Oak	*Quercus robur*	1151323	36	37	20	0.949	1	0.974
Worm	*Lumbricus terrestris*	3126801	17	28	15	0.903	1	0.949
Crab	*Callinectes sapidus*	312939	45	94	36	0.95	1	0.97
Spider	*Loxosceles reclusa*	1182604	17	27	16	0.871	1	0.931
Ciliate	*Paramecium aurelia*	484359	2	3	3	1	1	1
Mushroom	*Psilocybe cubensis*	190054	9	9	7	0.9	1	0.947
Beetle	*Oryctes nasicornis*	982103	2	5	5	1	1	1
Walrus	*Odobenus rosmarus*	328627	57	100	35	0.909	1	0.952
Water Bear	*Hypsibius dujardini*	1053826	4	5	5	0.833	1	0.909
Virus	Tobacco mosaic virus	8615186	2	5	4	1	1	1
Copepod	*Eurytemora affinis*	1020941	6	6	4	1	1	1
Kelp	*Nereocystis luetkeana*	902899	9	7	5	0.875	1	0.933
Tube Worm	*Riftia pachyptila*	393274	55	68	17	0.523	1	0.687
Honey Bee	*Apis mellifera*	1045608	61	222	54	0.978	1	0.989
GRAND TOTAL			487	1038	362	0.889	1	0.941

### Description of text

The text data objects used in this study were accessed through the EOL API. EOL accesses data objects from its 250 content partners that deliver structured data so that information from many sources can be integrated on an EOL page based on taxon and subject. The text data objects used in this study were written for a general audience and vary in length from one to 12,075 words. All text data objects used in this study were in English and labeled as “Trusted” by EOL curators. Content partners include, but are not limited to, Wikipedia (http://wikipedia.org), Animal Diversity Web (http://animaldiversity.org) and ARKive (http://www.arkive.org).

### Data retrieval

EOL exposes their content via an API that serves data in XML or JSON. Every taxon and data object in EOL has its own identifier which can be used to call it and its associated metadata through the API. EOL updates content from hundreds of providers every week, so interacting with EOL content through the API ensures relevance. API documentation can be found at http://eol.org/info/api_overview.

### Building the dictionary

Text data objects connected to each of the test species were read and manually annotated for ecologically relevant words and phrases. These words and phrases were placed in a dictionary as keys and the appropriate DBpedia URIs as the corresponding values. For example, ‘uterine cannibalsm’: ‘http://dbpedia.org/resource/Oophagy’ is included in the dictionary. DBpedia URIs were collected using the DBpedia Keyword API and manually placed in the dictionary. The documentation for this API can be found at http://wiki.dbpedia.org/lookup/. Any URIs can be placed in the dictionary (or removed) to suite user need.

An important issue for dictionary creation is how much of and which word or phrase to include. For example ‘predator’, ‘predation’, ‘predatory’ are all words used to describe a predator prey interaction that we would want tagged with the DBpedia URI for Predation (http://dbpedia.org/resource/Predation). This can be handled by having three separate keys in the dictionary with identical values or by having one key, ‘predat’, that will find all of the words. We used the latter strategy to handle plurals and any other form of a word/phrase. This reduces the size of the dictionary and thus the time it takes to build it and iterate over it in the workflow. This strategy would generate many false positives if applied to general text, but in the biological literature chances of encountering “predat” outside the context of predation are smaller.

### URI annotation

One code module was written to annotate EOL text objects with relevant DBpedia URIs (https://github.com/EOL/pseudonitzchia). The module used a list of Data Object IDs to call the API and get a JSON response. Each text data object was converted to lowercase. This helped control the size of the dictionary by not requiring separate entries for ‘prey’ and ‘Prey’. The module then went through each key in the dictionary and searched for it in the data object text returned in the JSON response. If the key was found, the module returned the corresponding value (the DBpedia URI) and moved on to the next key. If the key was not found, the module returned nothing and moved on to the next key.

### Information extraction and network building

The important information extraction task for this project was to generate a species association network using information in EOL text objects. Another code module was written to call the EOL API using EOL taxon IDs and retrieve the text objects under the “Associations”, “Trophic Strategy” “General Ecology” and “Habitat” subchapters (https://github.com/EOL/pseudonitzchia). We focused on these subchapters so we could extract information from text specifically describing ecological interactions (see discussion). The JSON response was parsed and cleaned using Beautiful Soup (http://www.crummy.com/software/BeautifulSoup/) and then passed to the GNRD API. GNRD (Global Names Recognition and Discovery) is a tool for finding scientific names in web pages, pdfs, Microsoft Office documents, images or freeform text using the TaxonFinder and NetiNeti name finding engines [30], [31]. Documentation for the GNRD API can be found at http://gnrd.globalnames.org/api. GNRD has a “resolver” function that is capable of reconciling multiple names for one species to a single, current name based on a user-chosen authority. For this project we chose EOL as the authoritative source of names and synonyms. More information about the resolver tool can be found at http://resolver.globalnames.org/. If a name was found on a page that, according to EOL, was an old name, the API returned the EOL ID of the new name. The extracted associations were returned in the format of Taxon A (Subject of Taxon page): Taxon B (Mentioned on Taxon page). Thus, if a taxon was mentioned on a page it was considered associated with the topic of the page. The data, in tabular form, was imported into Cytoscape for visualization and analysis [32]. In the network visualization, the nodes represent taxa and the edges represent an interaction between the taxa.

### Metrics testing and analysis

#### URI annotations

A human annotator read all of the text objects for the test species and annotated each of them with appropriate DBpedia URIs. Precision, recall and F1 Score were calculated for each test species and overall (Table 1). Errors were noted and categorized (Table 2). Precision was calculated as the URIs correctly assigned divided by the total number of URIs assigned to a text object. Recall was calculated as the URIs correctly assigned divided by the total number of URIs that were supposed to be assigned. The F1 Score is an overall measure of accuracy and is the harmonic mean of precision and recall. It is calculated by dividing precision by recall, then dividing that quantity by the sum of precision and recall, then multiplying that quantity by 2.

**Table 2:** URI Annotation Errors.

Species	Negation	Describing Related Taxa	Word Part	Generalities	Homonym	Total Errors
Shark	2	7	1	2	0	12
Lion	0	4	2	0	0	6
Moss	N/A	N/A	N/A	N/A	N/A	N/A
Diatom	0	1	1	2	0	4
Mosquito	0	0	0	0	0	0
Bacteria	0	1	1	3	1	6
Cactus	0	0	0	0	0	0
Oak	0	2	0	0	0	2
Worm	0	1	0	1	1	3
Crab	0	3	0	0	1	4
Spider	0	1	0	0	2	3
Ciliate	0	0	0	0	0	0
Mushroom	0	0	0	1	0	1
Beetle	0	0	0	0	0	0
Walrus	1	0	2	1	3	7
Water Bear	0	0	0	0	1	1
Copepod	0	0	0	0	0	0
Virus	0	0	0	0	0	0
Kelp	0	0	1	0	0	1
Tube Worm	1	1	1	0	1	4
Honey Bee	0	0	2	0	1	3
GRAND TOTAL	4	21	11	10	11	57

#### Associations network

Three human annotators with biodiversity expertise read all of the text objects for the test species and developed a list of association statements representing real ecological relationships between taxa. Taxonomic relationships were not included. If the text said that a species was, for example, pollinated by members of a family, the association was recorded as one between a species and a family, not as several between that species and every member of that family. Some annotators worked before the automatic process and others worked after, but in all cases the annotators had no prior knowledge of the algorithm results. Annotator agreement was calculated using Fleiss' Kappa [33]. The workflow result was evaluated against performance of the GNRD API working directly on the EOL taxon page without the intervention of our workflow (See Appendix S1 baseline.txt). Precision, recall and F1 Score were calculated for the baseline result (GNRD alone) and the workflow result compared to a human-created “gold standard” (See Appendix S1 gold_standard_int.txt). Precision was calculated as the interactions correctly extracted by the workflow divided by the total number of interactions the workflow extracted. Recall was calculated as the interactions correctly extracted by the workflow divided by the total number of interactions that were supposed to be extracted. The F1 Score was calculated in the same way as for the URI annotations. When constructing the network by hand, the annotator must not only look for terms that refer to taxa, but make a judgment call as to whether or not the author is describing an interaction. We did not want to include taxa that were mentioned solely for comparative purposes, for example. The annotator-created and automated networks were visualized using Cytoscape software [32].

## Results

### URI annotations

The workflow successfully annotated 487 text data objects associated with 21 species in EOL using a biologically-focused dictionary with 239 ecologically relevant entries (See biodict.txt in Appendix S1). Some of those text objects did not receive any annotations (14%). The most annotations that any one text object received was 33 and the average number of URIs assigned to a text object was 2.1.

The performance metrics for this particular method depended almost entirely on the construction of the dictionary. Overall, 80% of the URI annotations were correct. Precision = 0.889, recall = 1, and the F1 Score = 0.941 (Table 1). The lowest F1 Score (0.687) was for the tube worm, *Riftia pachyptila*. Recall is perfect, indicating the ability of the computer to find strings in text, a simple task. False positives decrease the precision and, in our data set, can be caused by several factors (Table 2). We have divided all of the errors into five categories:

1. Negation - This workflow cannot distinguish between “This species migrates.” and “This species does not migrate.” Both texts would be annotated with the URI for migration. Negation is a common NLP problem with rules-based solutions that rely heavily on context [34]. Coping with negation in biodiversity and ecology text is possible, but would require additional analysis.
2. Describing related taxa - Some text objects will use terms describing a related species that will cause a text object to be annotated with a URI that does not apply to that taxon. One example found on the Great White Shark page is the text “white sharks often attack dolphins and porpoises from above, behind or below to avoid being detected by their echolocation”. The text object containing that line would be annotated with the URI for echolocation, even though sharks do not have echolocation. However, since sharks have to cope with echolocation in their prey species, one could argue that this annotation would be relevant. For the metrics calculations in this publication, we took the conservative approach and did not consider them relevant.
3. Word or phrase part - Some terms will occur within other terms that mean something totally different. For example, the term “forest” in “kelp forest”. This will result in an oceanic species being annotated with a URI for a terrestrial habitat. This error also occurs if a content provider's name appears in the text object and contains a relevant term like “garden” or “marine”.
4. Generalities - Some terms are used in a general sense, not specifically referring to the taxon of interest and may cause a text object to be annotated with an irrelevant URI. This often occurs in headings. For example, a text object could give examples of why a species would migrate as an aside “e.g., to breeding or wintering grounds, to hibernation sites”. This would result in the text object being annotated with the URI for hibernation even if the species does not hibernate.
5. Homonym - Sometimes the same term can have a subtly different meaning in different contexts. The available URIs or the dictionary may not have the appropriate level of specificity. For example, the DBpedia URI for migration refers specifically to bird migration and thus cannot be used for any other group. The DBpedia URI for molt refers specifically to arthropod molting and cannot be used for bird molting. The term “attack” can describe an act of predation, self defense or disease, so it is not clear which URI to use.

The largest problem in our data set is the “describing related taxa” problem (37% of errors) followed by the “word part” and “homonym” problem (each are 19% of errors). Negation is the least of our problems (7% of errors).

### Association network

**Figure 1:**
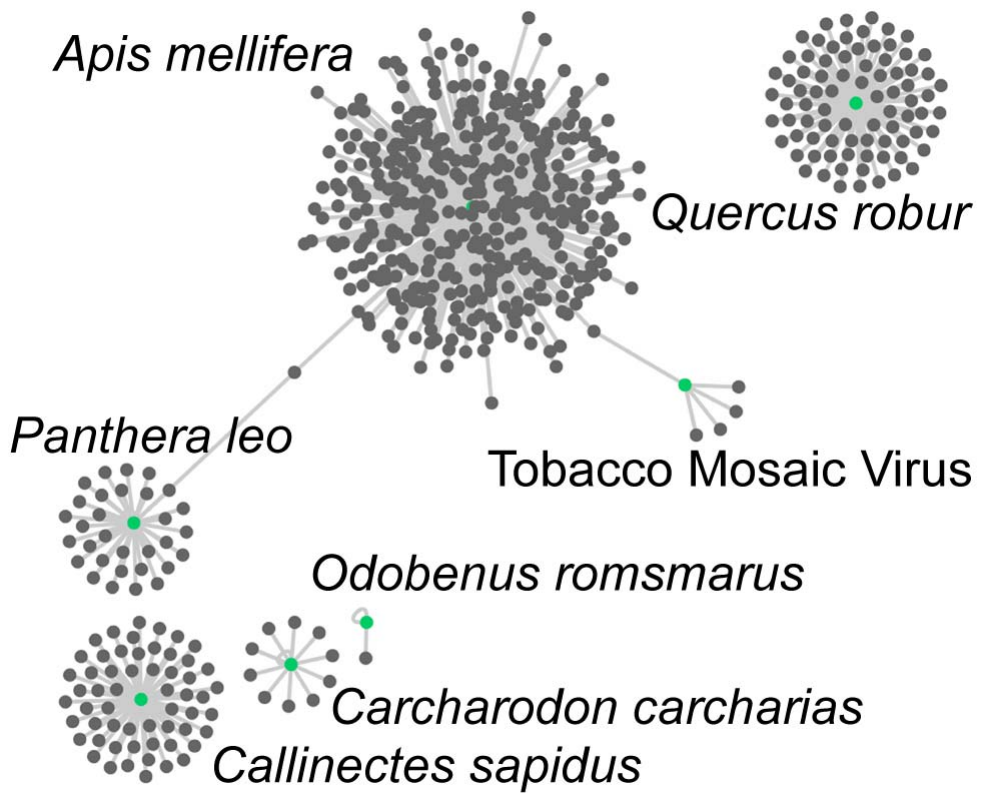
Automated Species Interactions. Taxon interaction network generated algorithmically by extracting information from text under the “Ecology” Chapter using the EOL and GNRD APIs. These associations are from the test species only.

**Figure 2:**
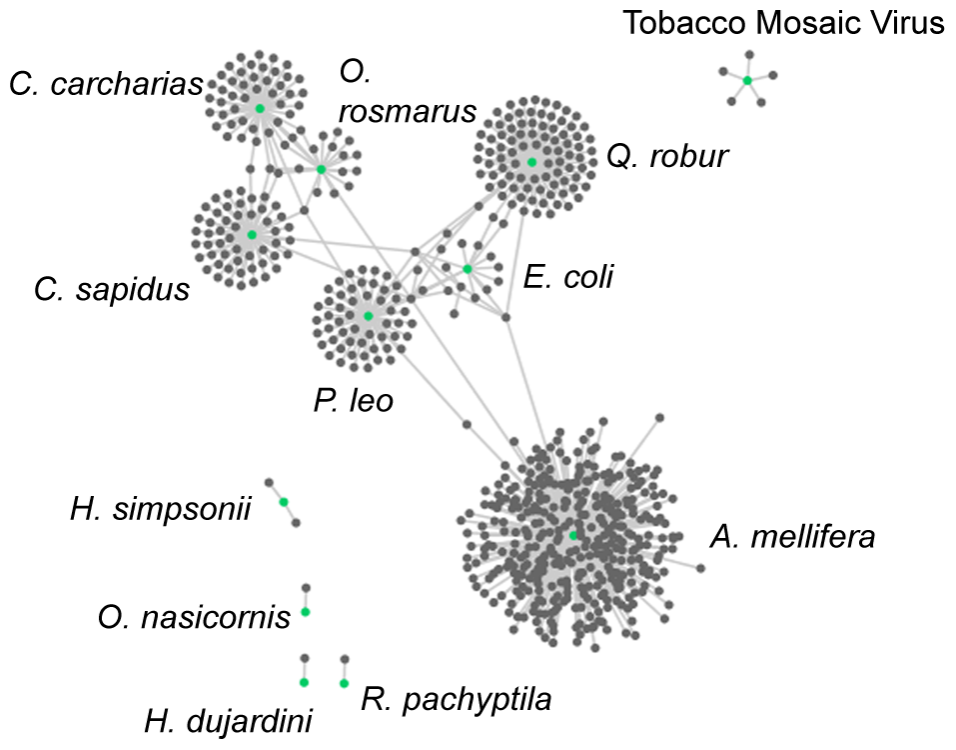
Manual Species Interactions. Taxon interaction network generated manually by reading through all of the text on an EOL taxon page and collecting scientific and common names. These associations are from the test species only.

We used the EOL and GNRD APIs to locate ecologically relevant text on a species page and find scientific names in that text. The output from this process was imported into Cytoscape [32] for visualization (Fig. 1). Our automated methods found 585 relationships between 581 taxa just by looking at the content under the “Ecology” Chapter of the 21 test species pages. A manual construction of the associations network using human annotation of the test species found 675 relationships between 657 taxa (Fig. 2; includes references made using scientific and common names). Agreement between annotators was 0.840 (Fleiss' Kappa; See Appendix S1, annotator_agreement.xlsx). Our automated methods had an overall precision of 0.844, a recall of 0.930 and an F1 Score of 0.885 (Table 3).

**Table 3:** Performance Metrics for Associations Workflow.

	True Positives	False Positives	False Negatives	True Negatives	Precision	Recall	F1 Score
*Carcharodon carcharias*	9	2	2	23	0.818	0.818	0.818
*Panthera leo*	30	2	4	16	0.938	0.882	0.909
*Hypnum fauriei*	0	0	0	1			
*Pseudo-nitzschia australis*	0	0	0	3			
*Culex pipiens*	0	0	0	6			
*Escherichia coli*	0	0	1	119			
*Harrisia simpsonii*	1	0	0	5	1.000	1.000	1.000
*Quercus robur*	81	2	3	15	0.976	0.976	0.976
*Lumbricus terrestris*	0	0	2	3			
*Callinectes sapidus*	49	9	11	13	0.845	0.875	0.860
*Loxosceles reclusa*	0	0	0	11			
*Paramecium aurelia*	0	0	0	1			
*Psilocybe cubensis*	0	0	0	51			
*Oryctes nasicornis*	0	0	1	24			
*Odobenus rosmarus*	1	1	1	17	0.500	0.500	0.500
*Hypsibius dujardini*	0	0	0	7			
Tobacco mosaic virus	5	0	1	0	1.000	0.833	0.909
*Eurytemora affinis*	0	0	0	4			
*Nereocystis luetkeana*	0	0	0	2			
*Riftia pachyptila*	0	0	0	7			
*Apis mellifera*	318	75	17	19	0.809	0.952	0.875
TOTAL	494	91	43	347	0.844	0.930	0.885

The workflow (using the GNRD API) found 585 taxon names and 494 of those represented ecological species interactions (84%). Our results had a total of 91 false positives and 43 false negatives. The largest source of false positives was the inclusion of higher taxon names for species that were mentioned (89%). For example, if a species and the Family it belongs too are both mentioned in a text object the algorithm will include them both, even though the interaction is with the species, not the entire Family. The largest source of false negatives was interactions discussed in text objects that were not under the Ecology Chapter and thus not analyzed (46%). Other important sources of error include formatting confounding GNRD (especially virus nomenclature, 14% of false negatives) and self referencing (7% of false positives). Out of the 583 unique names GNRD found, 36 (6%) were identified as an “old” name and needed to be reconciled to the new name.

For the task of identifying ecological interactions in text, GNRD alone had a precision of 0.477, a recall of 0.957 and an F1 Score of 0.636 (Table 4). The workflow, which makes use of GNRD, was much more effective at extracting ecological interactions, with a relative improvement in the F1 Score of 39% over the baseline.

**Table 4:** Performance of GNRD without Associations Workflow.

	True Positives	False Positives	False Negatives	True Negatives	Precision	Recall	F1 Score
*Carcharodon carcharias*	10	37	1	0	0.213	0.909	0.345
*Panthera leo*	33	45	2	0	0.423	0.917	0.579
*Hypnum fauriei*	0	2	0	0			
*Pseudo-nitzschia australis*	0	7	0	0			
*Culex pipiens*	0	7	0	0			
*Escherichia coli*	0	135	1	0			
*Harrisia simpsonii*	1	7	0	0	0.125	1.000	0.222
*Quercus robur*	82	26	2	3	0.759	0.976	0.854
*Lumbricus terrestris*	2	4	0	0	0.333	1.000	0.500
*Callinectes sapidus*	53	28	2	1	0.654	0.964	0.779
*Loxosceles reclusa*	0	16	0	0			
*Paramecium aurelia*	0	3	0	0			
*Psilocybe cubensis*	0	55	0	1			
*Oryctes nasicornis*	1	25	0	0	0.038	1.000	0.074
*Odobenus rosmarus*	2	29	0	1	0.065	1.000	0.121
*Hypsibius dujardini*	0	8	0	0			
Tobacco mosaic virus	5	3	1	0	0.625	0.833	0.714
*Eurytemora affinis*	0	6	0	0			
*Nereocystis luetkeana*	0	4	0	0			
*Riftia pachyptila*	0	12	0	0			
*Apis mellifera*	321	101	15	0	0.761	0.961	0.849
TOTAL	510	560	24	6	0.477	0.957	0.636

## Discussion

### URI annotations

The performance of the URI annotation workflow depended entirely on the contents of the dictionary; building this dictionary was a manual process. This type of dictionary matching method has limited utility when the dictionary is not complete relative to user need. This problem can be partially addressed through methods currently employed by community ontology projects. For example, terms and their corresponding URIs in an ontology can be automatically formatted into a dictionary. Many ontologies are curated by experts who add new terms, reconcile synonyms and add connections to other knowledge bases. One could leverage Uberon, a multi-species anatomy ontology containing over 10,000 terms [35] that can be easily transformed into a dictionary and used to annotate text based on morphology.

A user can run the program with whatever dictionary he/she chooses to target an area of interest. This strategy can be further improved by focusing annotations on taxa rather than text objects and thereby requiring less specificity in the dictionary. For example, if the goal were to annotate *Panthera leo* (EOL ID 328672) with the URI for predation, rather than every text object that discusses predation, we can be much more general with our dictionary key values. We only need “predat” instead of also needing “attack” “feed on” and “hunt” which can describe interactions not related to predation. Automating dictionary creation may also speed the process, but this would entirely depend on user need. For example, if a user were only interested in one key term, automation would not be helpful.

**Figure 3:**
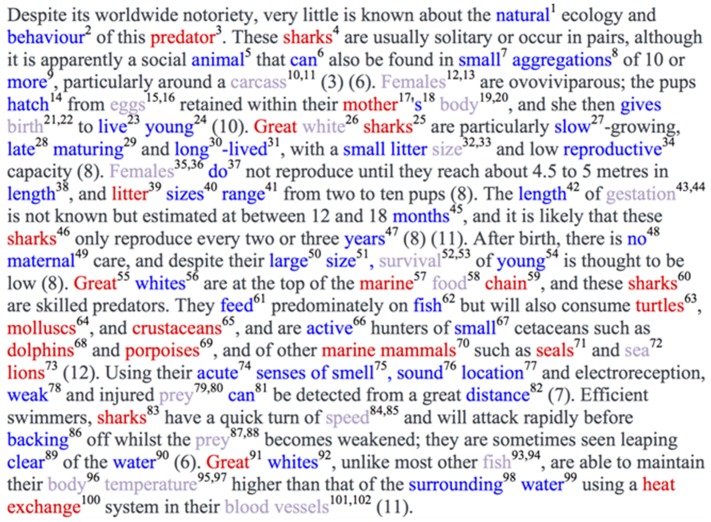
Annotator Comparison. Combined results from Terminizer (blue) and DBpedia Spotlight (red) when given an EOL text object to annotate (originally from ARKive). Strings that were annotated by both tools are colored purple. The superscripts correspond to the Identifier column in Table S1.

There are many existing tools that can add semantic annotations to text. Many of these have been tested and developed using news articles and other non-specialized text [36], [37]. We have explored two annotators within the context of ecology text: Terminizer [38] and DBpedia Spotlight ([39]; Fig. 3, Table S1). Terminizer annotates text when it finds strings that match ontological terms from 40+ biological ontologies in the OBO Foundry. DBpedia Spotlight annotates text with DBpedia URIs when it finds a relevant string. To explore these systems, we submitted a single text object to Terminizer and DBpedia Spotlight (Fig. 3). Terminizer made 65 annotations from 21 different ontologies. Most of the annotations Terminizer made were correct (40%), but there were still large numbers of incorrect annotations (37%) and we were not able to verify some annotations (25%) because the terms were not defined in their respective ontologies. DBpedia Spotlight made 36 annotations, all with DBpedia URIs. Most of these annotations were correct (70%) and only one annotation could not be verified as correct or incorrect (<1%). The errors made by Terminizer were mostly due to specificity and domain. For example, some annotations were made that described unrelated model organisms or molecular interactions. Another common error included annotating only one word in a two-word term, like “litter size” and “food chain”. DBpedia Spotlight was able to find two-word terms like “food chain” and “marine mammal”, but had disambiguation problems with terms that also had a pop culture reference. For example, “carcass” was annotated with a URI for a music band and “seal” was annotated with the URI for the Unites States Navy SEALs. The performance of these annotators may be improved by restricting the ontologies they are using. Terminizer has an option to choose specific ontologies in OBO or insert your own (in OBO format). DBpedia Spotlight has settings that can allow for refinement or it can take your own dictionary, if you can set up your own instance. Our method annotated the same text with six URIs from our dictionary, including “social animal”, “electroreception”, “gestation”, “ovoviviparous”, “predation” and “marine”. These terms represent our ecological knowledge annotation goal. As expected, the tool we designed ourselves best addressed our needs. Only half of these desired terms were found by Terminizer and/or DBpedia Spotlight: “gestation” was found by both and “predator” and “marine” was found by DBpedia Spotlight. Our tool avoided some disambiguation problems by only working with biological text (so we don't have to worry about pop culture references like DBpedia Spotlight) and carefully selecting the terms in the dictionary. For example, “ovoviviparous” has a single meaning. A possible next step would be to fashion either a dictionary or an ontology file containing our terms so that Terminizer or DBpedia Spotlight could be used as an attractive user interface. We would like to suggest that annotators include pathways for users to add terms of interest within the functionality of the interface.

### Association network

The ability to build an automated taxon associations network from narrative depends on three factors 1) the ability to recognize taxon names in text, 2) the ability to reconcile multiple terms used for the same species and 3) the ability to determine if a taxon mention refers to an ecological interaction.

Recognizing taxon names in text is a major area of research. Several tools have been developed that can find scientific names [5] and we use one of them, GNRD, in this project. None of these tools can find common names in text, which results in missing approximately 13% of the total associations and taxa. Some work has been done in the field of Earth Science to recognize common names for geomaterials using contextual clues with some success (Jenkins pers. comm.). EOL has a compilation of over 780,000 common names across the tree of life that are linked to an EOL ID. The EOL API can return an EOL taxon for a common name submitted, but recognizing a common name in narrative is still a major challenge. One possible strategy is to search for the common names corresponding to the found scientific names (Mozzherin pers. comm.), but that would not find taxa only mentioned by common name and would not improve the performance of the workflow.

One cannot assume that all of the taxa mentioned on an EOL taxon page have an ecological relationship with the topic of that page. Taxa are mentioned for several reasons including comparison, taxonomic relationships and in discussion of a common phenomenon, like poisonous mushrooms. Users must balance their need for precision and recall against the amount and type of text they process. As mentioned above, the largest cause of false negatives was names found in text objects that were not under the Ecology chapter. Most of those text objects were from Wikipedia. Including Wikipedia content in our analysis is likely to also dramatically increase false positives because Wikipedia text often includes a list of taxonomically related species and subspecies.

This annotation and information extraction exercise represents the initial explorations for making EOL data semantically available and creating a semantic infrastructure describing species interactions. Most available information about species is organized taxonomically or phylogenetically. EOL is an example of this, allowing users to browse information using a taxonomic classification. Using a taxonomic infrastructure, a user is not able to navigate from a lion directly to its prey, its parasites or its competitors. Long term goals include enabling an ecologically-focused browse capability in EOL and making EOL content semantically available. Our short term vision is that users who are learning about Oophagy on the Wikipedia page can easily find EOL text objects and taxon pages that discuss the phenomenon through the linking of DBpedia and EOL URIs.

These workflows are not perfect and, considering the complexity of biology and language, are not likely to ever be perfect. A text object might say “oak trees provide habitat for many animals”. Animals refers to Animalia, but the authors were not intending to say that all Animalia live in oak trees. A text object may use a high level taxon name that in practice only applies to a single species in a particular area. For example, referring to deer (Cervidae) on the US East Coast really only refers to White Tailed Deer (*Odocoileus virginianus*). Some terms are used as though they correspond to a taxonomic group, but in reality do not, such as seabirds and worms. From an EOL perspective, it is far more important to have high precision than high recall (Wilson pers. comm.). Fortunately, EOL has curators and staff to cope with the errors that may result. The algorithm may be accurate enough to reduce the manual work to a level that is reasonable for the curators and staff. There are approximately 1,200 EOL curators of which 182 are considered “active” and make approximately 1,500 actions per month. This has resulted in approximately 22,000 EOL pages that have been subject to one or more curatorial actions since the curatorial functions were enabled in 2008. Still, other methods of assisting users in avoiding bad text-mining results are recommended to any data consumer, including EOL, such as not importing until an average F1 Score threshold is met, assigning numerical ratings to datasets based on rate and type of errors, and flagging datasets that are the result of text-mining.

## Future Work

Using machines to extract knowledge from centuries of biological descriptive data is a promising strategy that still needs significant investment. Below we discuss future work specifically relating to knowledge extraction from sources like EOL.

1. *Classify associations using a well-developed interaction vocabulary*. An association network like the one developed in this project could be made even more informative if the association was defined more specifically. At the moment, the network only states that a relationship exists. No information is given about the nature of the relationship. The most efficient way to do this is to develop a standard vocabulary for ecological relationships that can be applied using clues from the surrounding text. A new generation of researchers is working on normalizing terms and relationships that can be applied to found associations [40].

2. *Inferring meaning with contextual clues*. Humans use context to cope with ambiguous terms. Computers can do this through distributional semantics. This is where meaning is inferred by quantitative analysis of word-associates using statistical methods such as Cosine Similarity, Jaccard Index and Maximum Entropy [41]. Algorithms exist that analyze text using distributional semantics and have been used successfully in the geosciences (Jenkins pers. comm.). We plan to apply these methods to EOL content. The URI annotations discussed in this paper can also provide contextual clues. For example, if a text object is annotated with the URI for predation and an association is also detected, a predatory relationship can be inferred. However, if multiple associations and multiple URI annotations are available for the same text object, it is unclear which goes with which. Development of these types of intelligence systems for ecological text is a step above the dictionary methods used here and represents our anticipated research trajectory.

3. *Scaling up*. There are two important ways to increase the speed of this process: improving data retrieval and removing as much manual intervention as possible. Considering the frequency with which EOL updates, interacting with content through the API is ideal; however, these types of requests are relatively slow. There may be small ways to improve efficiency of these methods in the future, such as not rerunning the algorithms over EOL content that has not changed, but increases in the speed of queries using an API will be limited. SPARQL queries, because of their bulk processing capabilities, have the potential to be a much faster way to interact with heterogeneous EOL data, but cannot be immediately implemented. Any manual step is a bottleneck, including the manual dictionary creation and the correction of errors. Scale is and will continue to be an issue for any project trying to work over the entirety of life and all of its data.

## Conclusions

Semantic strategies for representing knowledge are finding a practical foothold in biological research [42], [43]. The potential of semantics for revolutionizing the way biodiversity knowledge is recorded and data are shared and used is vast. The work described in this paper is a modest step toward large-scale knowledge extraction from an aggregator of biological text, EOL. As we continue to develop our workflows and data structures we will be increasingly able to utilize the advantages of semantics, including inferencing and reasoning.

## Supporting Information

Table S1 **Terminizer and DBpedia Spotlight Annotation Details.** Results from the annotation of an EOL text object with Terminizer and DBpedia Spotlight.
(TXT)

Appendix S1 **Data Sets.** List of data files available from this study and their description.
(PDF)
