# Supplementary material for: Knowledge Extraction and Semantic Annotation of Text from the Encyclopedia of Life
Source: PLoS One. 2014 Mar 3;9(3):e89550. doi: 10.1371/journal.pone.0089550 (PMC3940440; doi:10.1371/journal.pone.0089550)
Supplement: Appendix S1 — Data Sets. List of data files available from this study and their description. (PDF) [file pone.0089550.s002.pdf]

Appendix S1. Data Sets. List of data files available from this study and their description.

EOL Test Species List (EOL species list.txt)
http://dx.doi.org/10.6084/m9.figshare.760594
List of test species used in this analysis. The file is comma delimited. The first column is the species common name. The second column is the scientific name. The third column is the EOL ID.

Semantic Annotation of Selected EOL Text (all_uri.txt)
http://dx.doi.org/10.6084/m9.figshare.760596
Output from the eco_terms_all_json.py code module applied to the test species. The file is comma delimited. The first column is the data object ID. The second column is the term found from the dictionary. The third column is the DBpedia URI annotation.

Ecology and Evolution Dictionary (biodict.txt)
http://dx.doi.org/10.6084/m9.figshare.760597
Dictionary file used by eco_terms_all_json.py to annotate text objects with DBpedia URIs.

Annotated Species (uri_species.csv)
http://dx.doi.org/10.6084/m9.figshare.760598
The results of eco_terms_all_json.py with text object IDs replaced by corresponding Taxon IDs and duplication removed.

Species Associations (associations.csv)
http://dx.doi.org/10.6084/m9.figshare.760599
Output from names_json.py applied to the test species that is ready for upload into Cytoscape. The file is comma delimited. The first column is the scientific name for the taxon that is the topic of the taxon page. The second column is the corresponding EOL Taxon ID. The third column is the scientific name of the taxon mentioned in the text object. The fourth column is the corresponding EOL taxon ID.

Manually Extracted Species Associations (associations_manual.txt)
http://dx.doi.org/10.6084/m9.figshare.760600
Results from manual extraction of all taxon names (common and scientific) in any text object on a taxon page. The first column is the topic of the taxon page. The second column is the taxon mentioned in the text object.

Replacement Dictionary (replace_dict.txt)
http://dx.doi.org/10.6084/m9.figshare.760601
Dictionary file used by names_json.py in the replace_all function.

Species Associations (associations_all.txt)
http://dx.doi.org/10.6084/m9.figshare.760602
Output from names_json.py applied to every species in EOL. The first column is the Taxon ID for the subject of the taxon page. The second column is the Taxon ID for the taxon mentioned on the page.

Gold Standard Interactions (gold_standard_int.txt)

http://dx.doi.org/10.6084/m9.figshare.871539

Human-created data set used to measure the results of the workflow. This is the list of all interactions described using scientific names. The header row is the list of test species. Each column is a list of interactions mentioned on the EOL species page.

Gold Standard Names (gold_standard_all.txt)

http://dx.doi.org/10.6084/m9.figshare.871540

Human-created data set used to measure the results of the workflow. This is the list of all scientific names. The header row is the list of test species. Each column is a list of all the scientific names on the EOL species page.

GNRD Results (baseline.txt)

http://dx.doi.org/10.6084/m9.figshare.871541

The header row is the list of test species. Each column is a list of the results returned by GNRD. These results were obtained by giving GNRD the URL of the EOL species page.

Annotator Agreement (annotator_agreement.xlsx)

http://dx.doi.org/10.6084/m9.figshare.871543

Calculation of Fleiss' kappa

Workflow Results (workflow_results.txt)

http://dx.doi.org/10.6084/m9.figshare.871544

The header row is the list of test species. Each column is a list of the results returned by the workflow described in this paper.

Annotator 1 (annotator_1_sci.txt)

http://dx.doi.org/10.6084/m9.fighsare.871545

Results from annotator 1

Annotator 2 (annotator_2_sci.txt)

http://dx.doi.org/10.6084/m9.fighsare.871546

Results from annotator 2

Annotator 3 (annotator_3_sci.txt)

http://dx.doi.org/10.6084/m9.figshare.871547

Results from annotator 3
